# Supplementary material for: 3D printing by stereolithography using thermal initiators
Source: Nat Commun. 2024 Mar 13;15:2285. doi: 10.1038/s41467-024-46532-0 (PMC10937977; doi:10.1038/s41467-024-46532-0)
Supplement: Supplementary file 1 — Supplementary Information [file 41467_2024_46532_MOESM1_ESM.pdf]

Supplementary Information

3D printing by stereolithography using thermal initiators

Doron Kam[1], Omri Rulf[1], Amir Reisinger[1], Rama Lieberman[1], & Shlomo Magdassi[1]*

[1]The Institute of Chemistry, Hebrew University of Jerusalem, Jerusalem, 91904, Israel.
These authors contributed equally: Doron Kam, Omri Rulf.

*email: magdassi@mail.huji.ac.il

Supplementary Discussion: photothermal PI estimation cost

The retail cost described in the manuscript shows average prices for large quantities according to the global wholesale trade platform (alibaba.com, 30 May 2023). The following prices are:

$$SPS{\sim}0.04\frac{\$}{kg},\qquad BzO{\sim}1\frac{\$}{kg},\qquad I2959{\sim}30\frac{\$}{kg},\qquad LAP{\sim}100k\frac{\$}{kg}$$

Sodium persulfate (SPS), benzoyl peroxide (BzO), IRGACURE 2959 (I2959), lithium phenyl-2,4,6-trimethylbenzoylphosphinate (LAP).

For simplicity, all costs used for the discussion below (Supplementary Fig. 1 and Supplementary Table 1) were taken from the Sigma-Aldrich website (on 30 May 2023), reflecting an upper barrier of costs (the industrial market prices are much cheaper). The differences in large quantities market prices and small lab-scale sample prices are expected.

The costs of the thermal initiators are: SPS = 0.0512 \$ g^{-1} or BzO = 0.027 \$ g^{-1}. The cost of silver nanoparticles (AgNPs) required for each gram of SPS is ~0.09 \$. Therefore, the total cost per gram of the thermal initiator system is only ~0.14 \$. The estimated cost for synthesizing the proposed AuNR is about ~25 \$ for 1 g of thermal initiator.

Supplementary Fig. 1 summarizes the costs of various commercial photoinitiators (PIs), classified according to their maximum absorbance peak on a **semilog** graph. Two trends can be seen (1) as the wavelength increases, the cost of the PIs increases too (2) water-soluble PIs are much more expensive than solvent-soluble PIs. As seen, our proposed materials (marked in black) span across a wide range of wavelengths, from 530-1076 nm, independent of the absorbance peak or solubility. The cost of our initiator system is about an order of magnitude cheaper than the common PIs for aqueous systems. If regular, spherical nanoparticles are used for the range of 400-500nm (silver and gold), the cost will be much lower than that of the AuNR, thus further decreasing the gap in prices, including for non-aqueous systems.

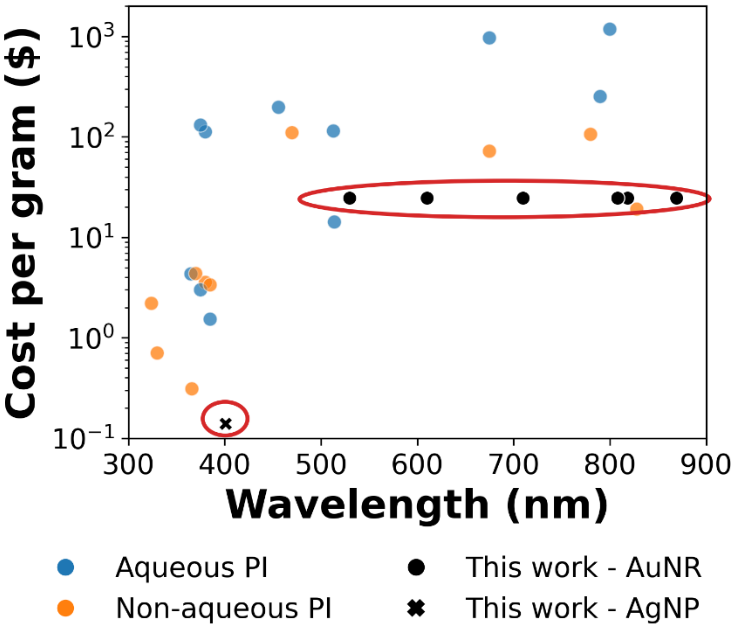

Supplementary Figure 1 | PI cost as a function of absorbance peak. The type of PI, for water or solvent, is marked in blue or orange, respectively. The black markings denote the current work of materials that can be soluble in both water and non-aqueous formulations; Black circles correspond to AuNR, and the black X symbol corresponds to AgNP (full details provided in Supplementary Table 1).

Supplementary Table 1. Summary of known PIs and their cost based on Sigma-Aldrich website (entered on 30 May 2023).

Name	Cost per gram ($)	λ_{max} (nm)	Absorbance spectrum (nm)	Solubility
**BzO**	**0.027**			**solvent**
**AuNR+BzO**	**24.68**		**530-1076**	**solvent**
**SPS**	**0.0512**			**water**
**AuNR+SPS**	**24.7**		**530-1076**	**water**
**AgNP+SPS**	**0.14**	**401**	**345-620**	**water**
TPO-NP	112.2	380	260-400	water
I2959	4.32	365	260-300	water
TPO	3.56	380	260-400	solvent
TPO-L	0.31	366	260-400	solvent
LAP	130.8	375	320-430	water
Ru(bpy)	197	456	400-500	water
369	2.2	324	260-400	solvent
Irgacure 819	4.36	370	260-400	solvent
184	0.704	330	200-380	solvent
BAPO-Oli	3	375	320-420	water
VA-086	1.53	385	260-430	water
Eosin-Y	14.2	514	470-540	water
BSEA	114.4	513	380-600	water
DETX	3.36	385	260-400	solvent
Bachl a	966	675	650-800	water
IR-140-BORATE	1180	800	700-850	water
iodonium salt	252	790	700-850	water
AlPc	72	675	650-850	solvent
IR-780 iodide	106	780	640-840	solvent
H-Nu 470	110	470	350-550	solvent
H-Nu 815	19.08	828	650-850	solvent

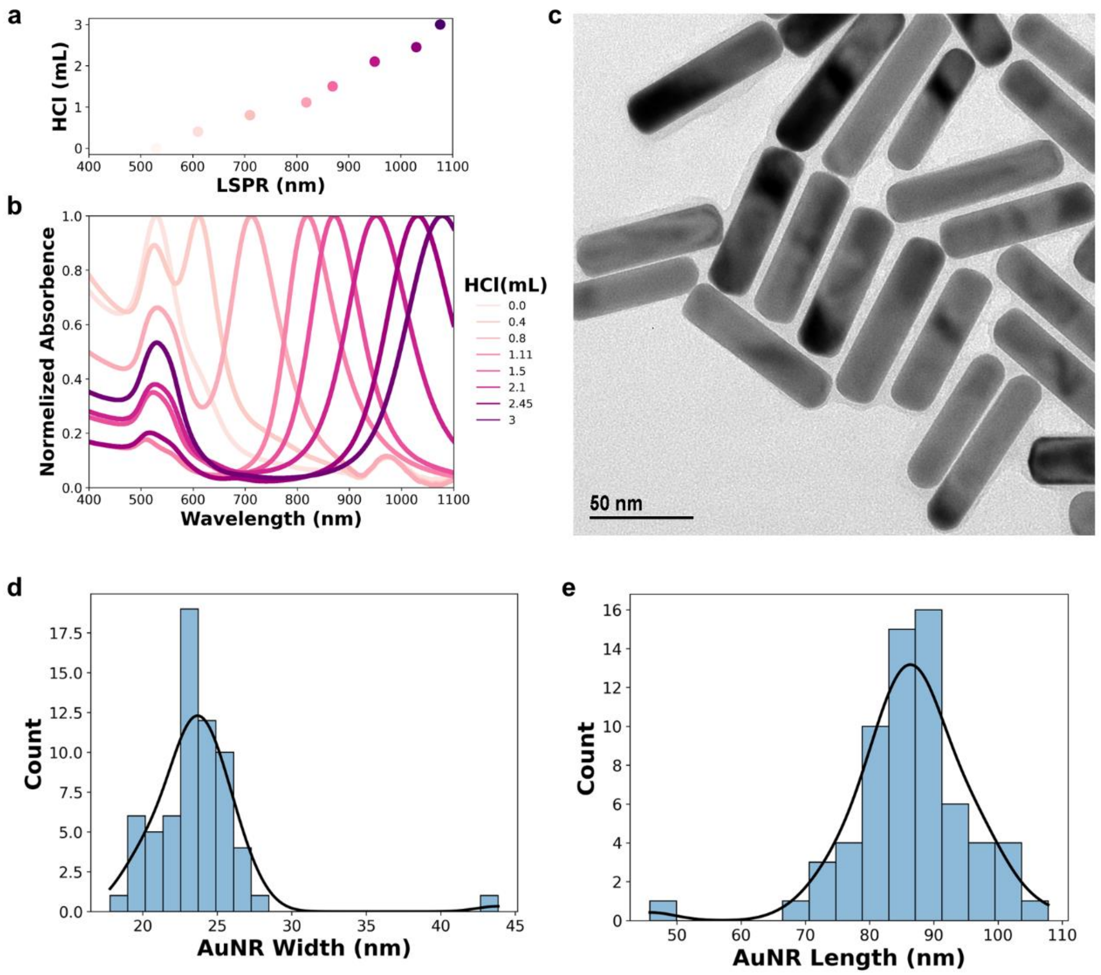

Supplementary Figure 2 | Adding different HCL volumes during the synthesis governs AuNR sizes, resulting in longitudinal surface plasmon resonance (LSPR) shifting toward longer wavelengths. a Volume of HCl added during the synthesis as a function of the LSPR absorption peak. **b** Normalized absorbance to the LSPR of AuNR as a function of wavelength. **c** TEM image of the 808 nm AuNR used in this work and the relevant particle analysis dimension of AuNR **d** width and **e** length.

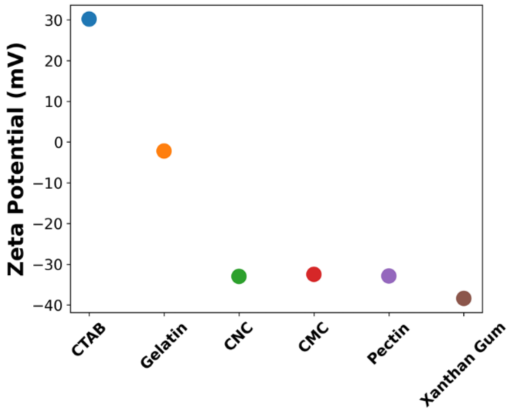

Supplementary Figure 3 | Zeta potential of ink compositions with various stabilizers.

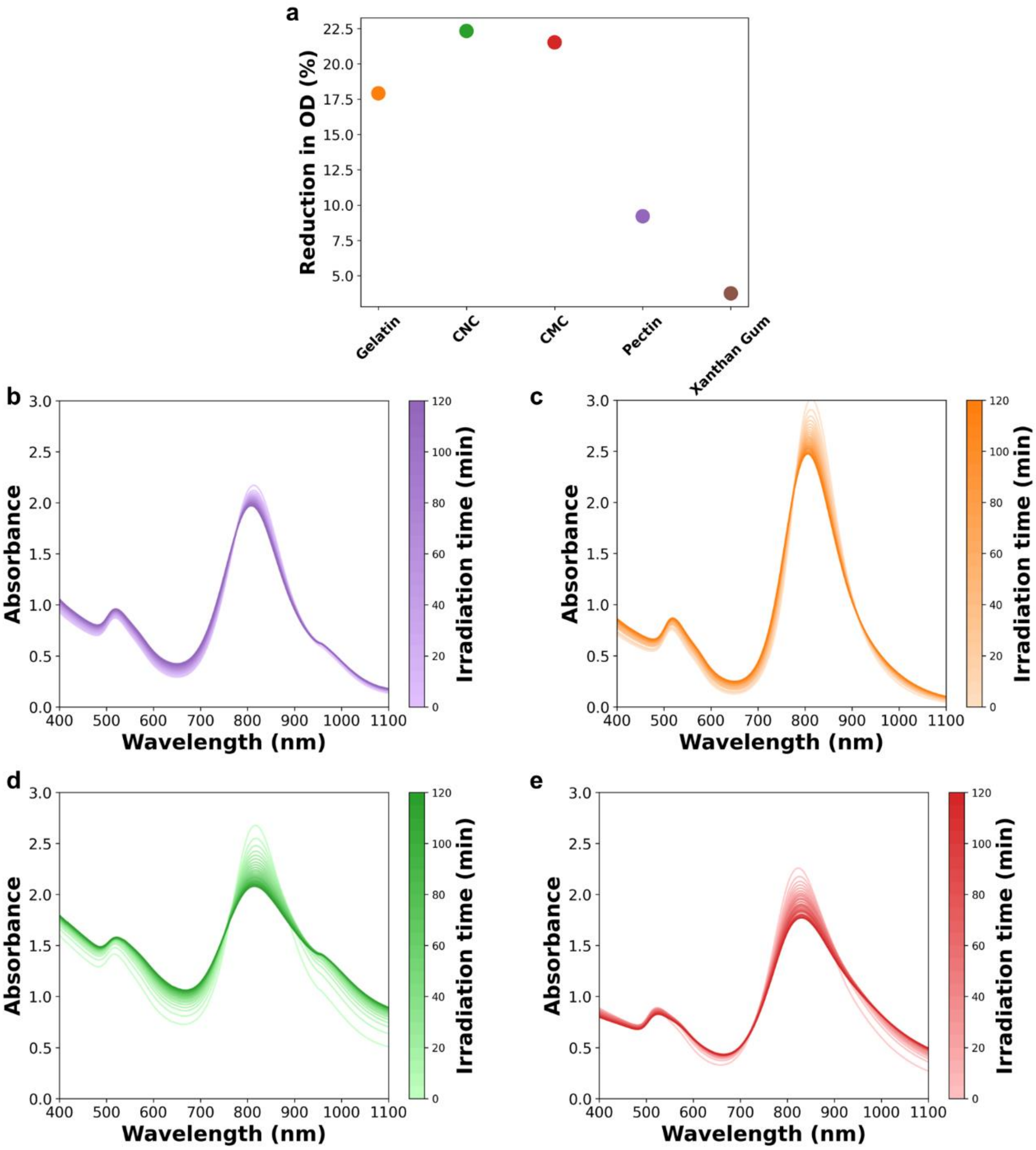

Supplementary Figure 4 | UV-vis spectroscopy for various stabilizers. a Reduction of the LSPR absorption optical density after two hours in the presence of different stabilizers. **b-e** Time-depended UV-vis spectroscopy of **b** pectin, **c** gelatin, **d** cellulose nanocrystal (CNC), **e** carboxymethyl cellulose (CMC).

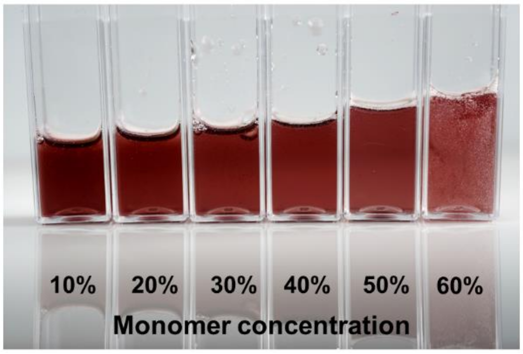

Supplementary Figure 5 | Ink composition with xanthan gum as a stabilizer with increasing monomer concentration. The monomer concentration (wt.%) includes both PEGDA+HEA, while the amount of initiator (AuNR+xanthan gum+SPS) remains constant. Visible aggregation in 60 wt.% monomers indicates the instability of the dispersion from this concentration and above.

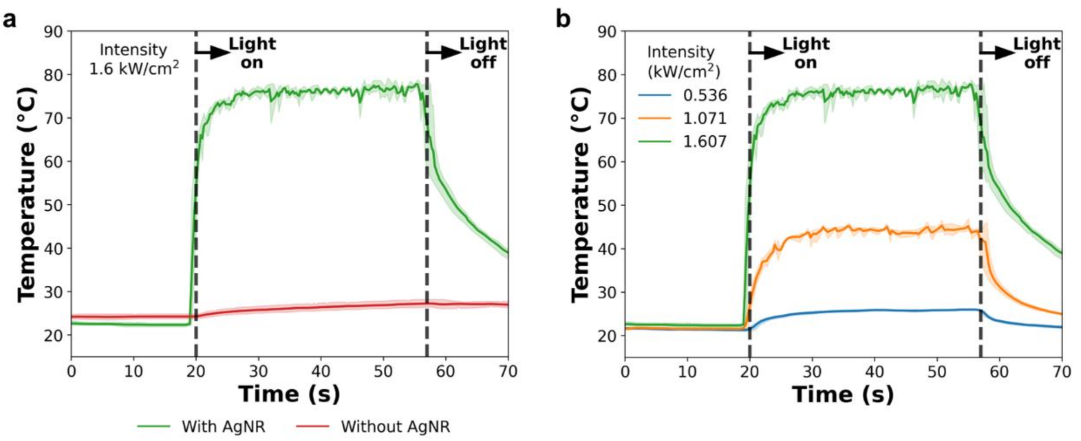

Supplementary Figure 6 | The role of the AuNR as photothermal converters by examining the effect of light irradiation on the ink temperature. a Ink composition temperature behavior with and without AuNR while irradiating 808 nm laser at 1.6 kW cm^{-2} intensity for a fixed place. **b** Ink composition temperature behavior with AuNR while irradiating 808 nm laser at 0.5, 1.1, and 1.6 kW cm^{-2} intensity for a fixed place.

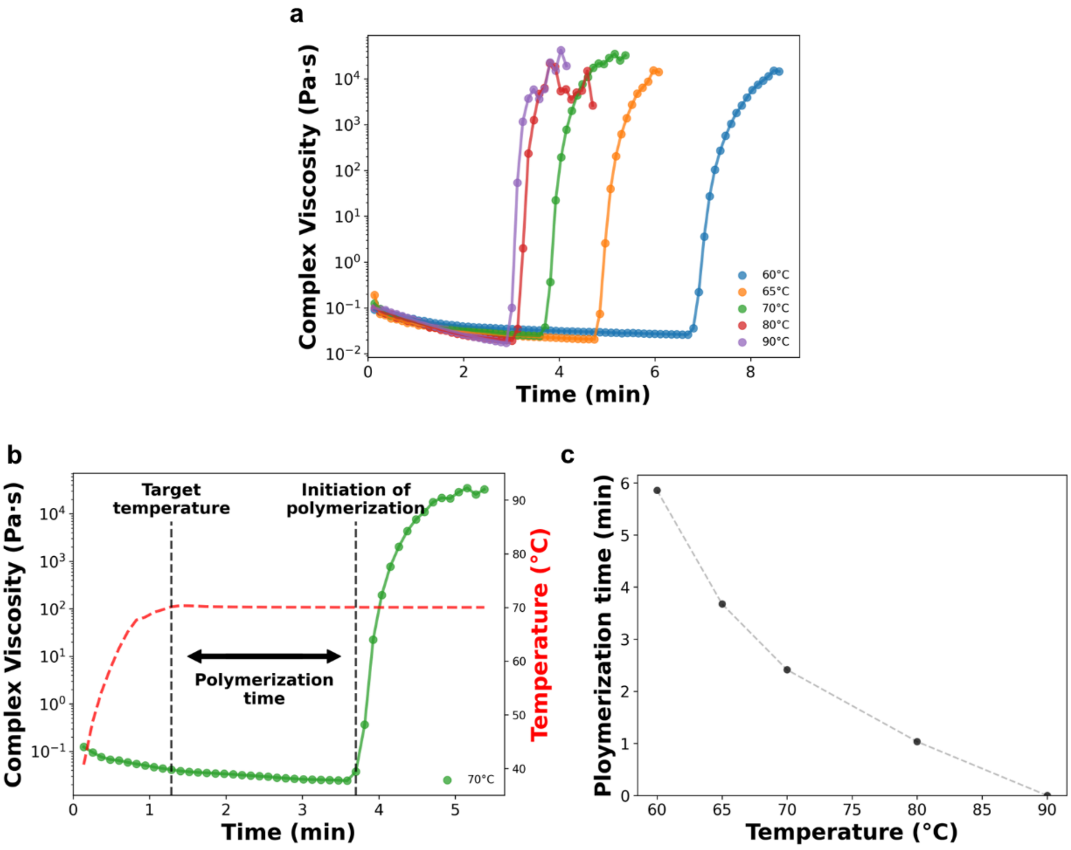

Supplementary Figure 7 | Dependence of polymerization time on the ink temperature by oscillatory rheology experiments for 1 wt.% SPS selected ink composition. a Parallel plate rheological measurements at f=0.5 Hz for different temperatures. **b** Example of polymerization time calculation for 70 °C. The target temperature is determined by the first time the desired temp is recorded, and the start of polymerization is determined with the increase in ink viscosity. **c** Overall polymerization time versus temperature.

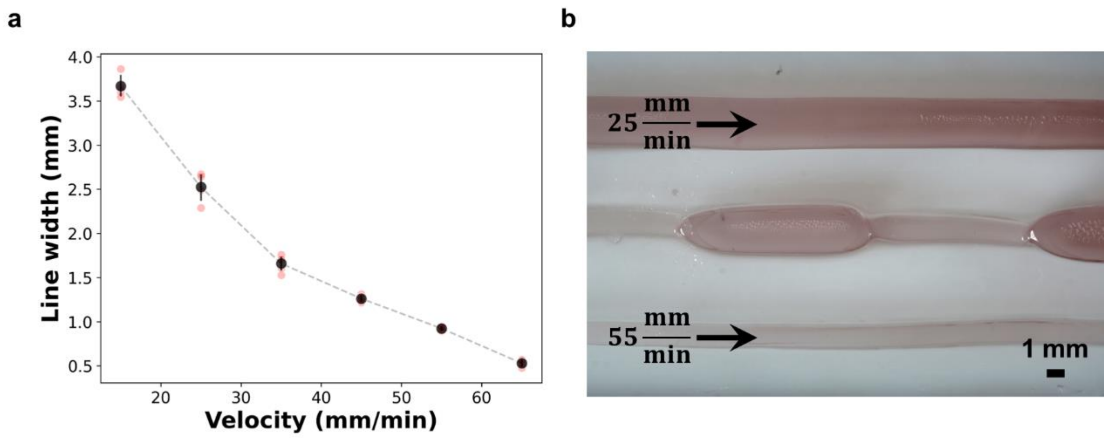

Supplementary Figure 8 | Line width measured for 2.1 kW cm^{-2} laser intensity at various velocities. a Line width measurements performed by microscope. Black markers represent averages from N=5, red markers indicate raw data, and error bars indicate 95 % CI. **b** Picture of three printed lines; above and below constant speeds of 25 and 55 mm min^{-1}, respectively, and a printed line at alternating the same speeds in the center.

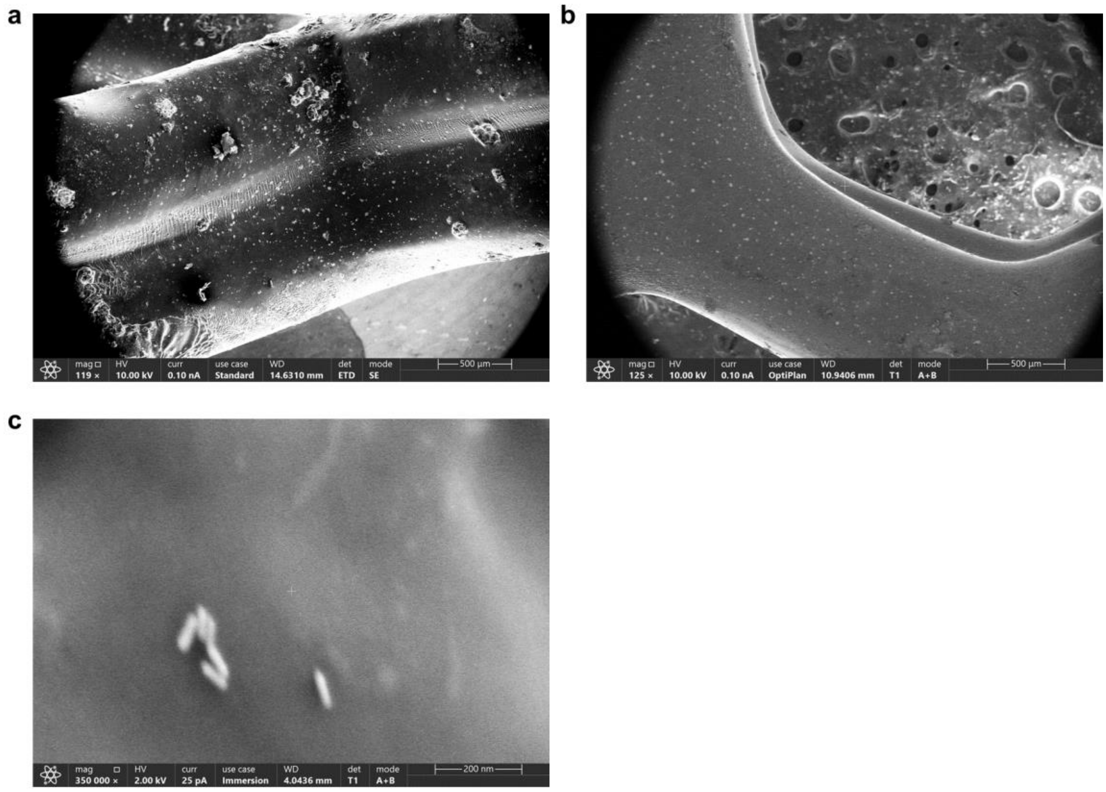

Supplementary Figure 9 | Raw SEM images of honeycomb hydrogel object used in Fig. 4. a Raw image of Fig. 4d. **b** Raw image of Fig. 4e. **c** Raw image of Fig. 4f.

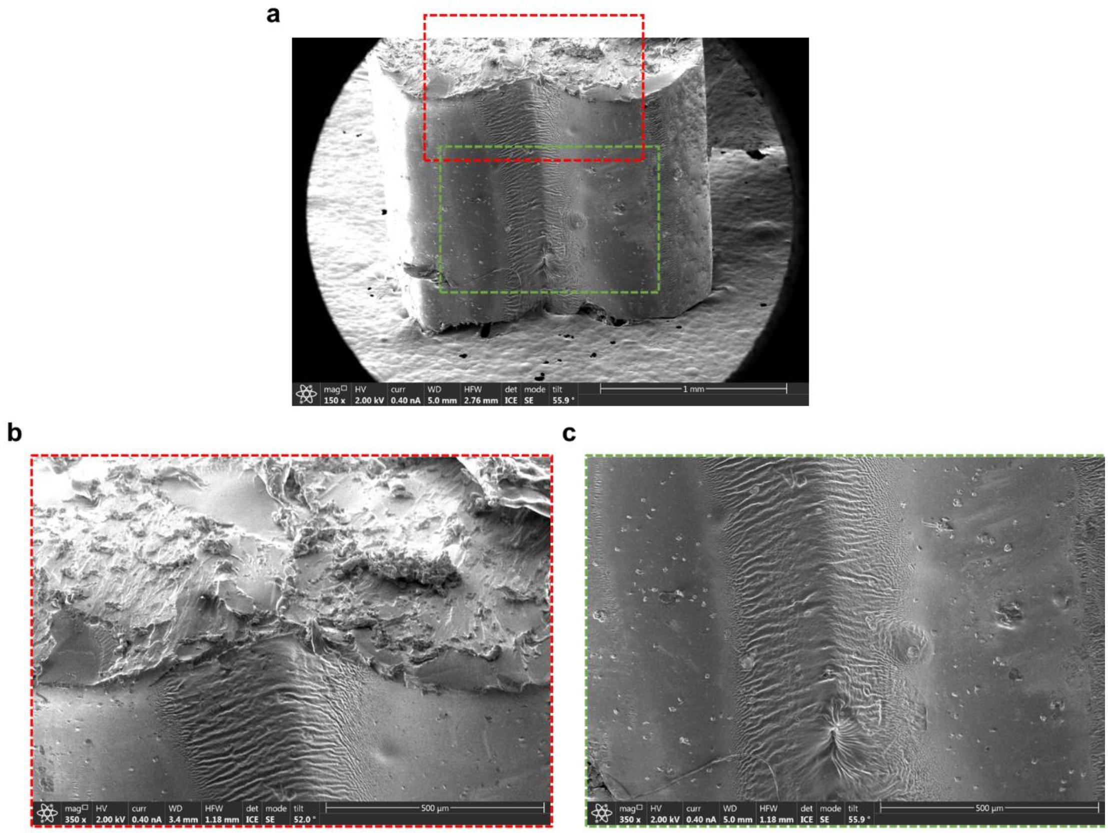

Supplementary Figure 10 | SEM images of infusion of two lines. a Long shot of two printed lines. **b** Close-up of cross-section cut showing full infusion of the two lines. **c** Close-up of surface infusion between the lines.

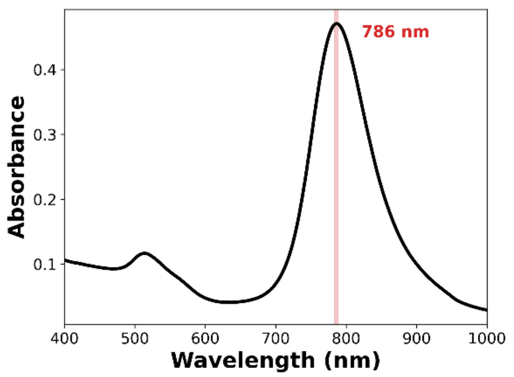

Supplementary Figure 11 | UV-vis spectrum for AuNR tuned to 780 nm for non-aqueous printing composition.

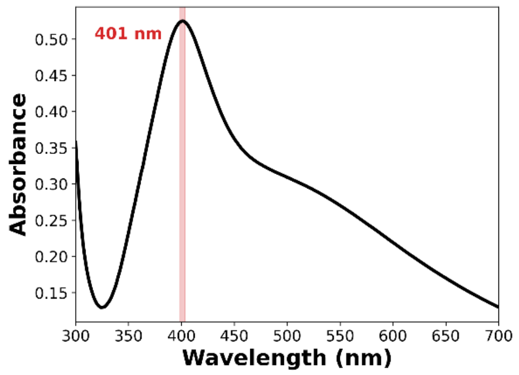

Supplementary Figure 12 | UV-vis spectrum for AgNP for aqueous printing composition.

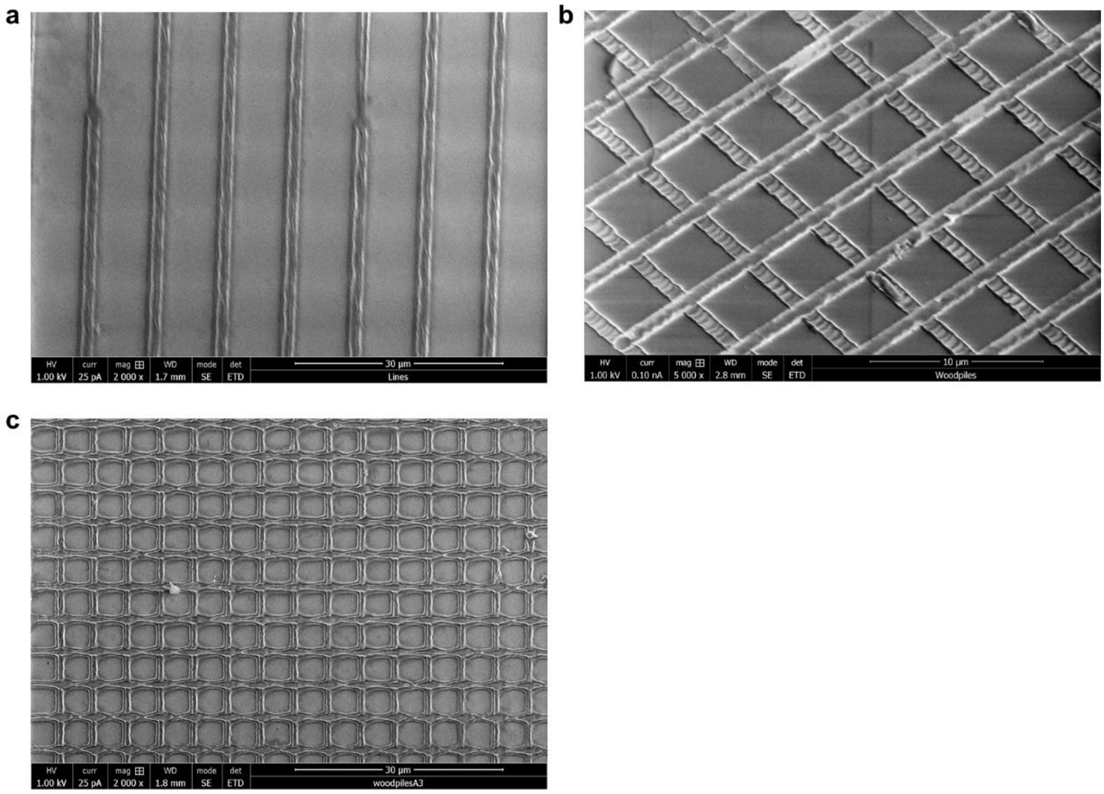

Supplementary Figure 13 | Raw SEM images of two-photons printing experiments using non-aqueous ink composition with NIR converters used in Fig. 5. a Raw image of Fig. 5a. **b** Raw image of Fig. 5b. **c** Raw image of Fig. 5c.

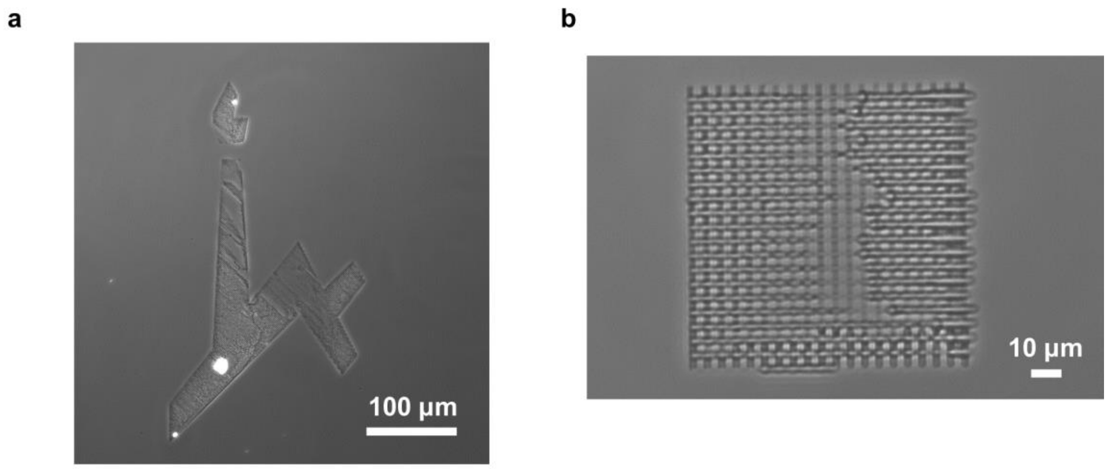

Supplementary Figure 14 | Light microscopy images of two-photons printing experiments using non-aqueous ink composition with NIR converters a HUJI symbol **b** woodpile.

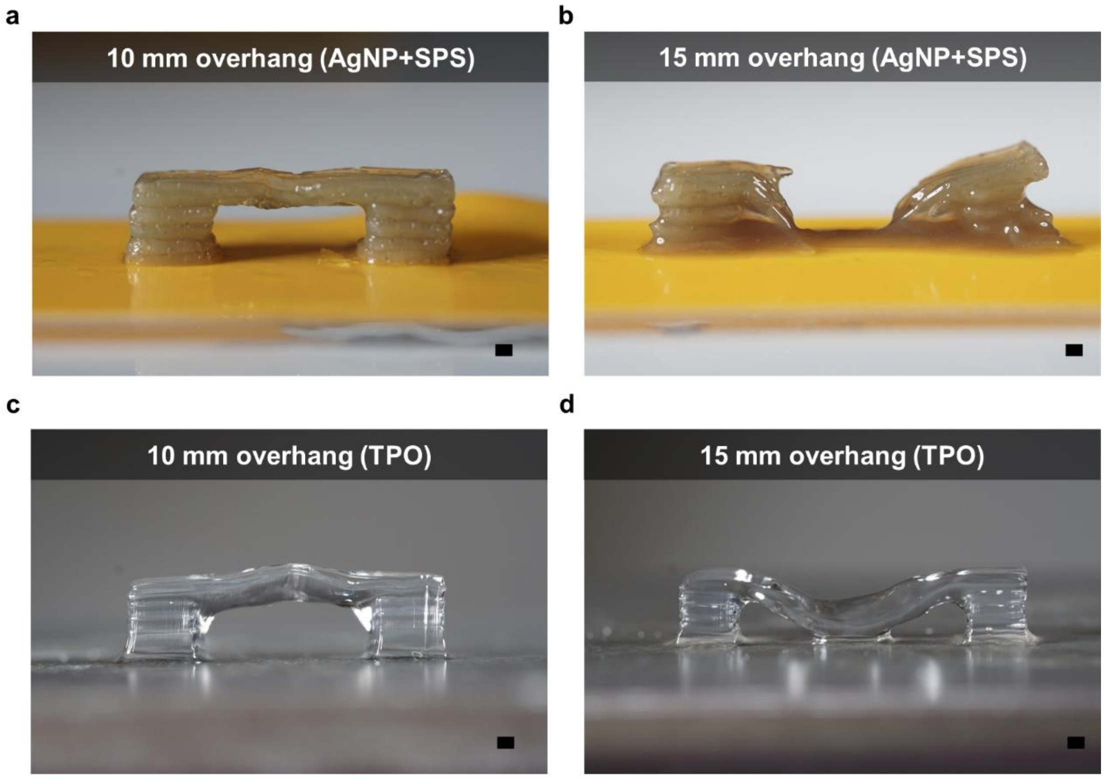

Supplementary Figure 15 | Overhang structure limitation printed with photothermal or photoinitiator. a-b Overhang structure printed by NIR-induced VPP using PEGDA+HEA monomer and AgNP+SPS initiator. **c-d** Overhang structure printed by digital light processing (DLP) using PEGDA+HEA monomer and TPO photoinitiator. Scale bars indicate 1 mm.
